# Cyclophosphamide post-haploidentical stem cell transplantation experience in an infant with IPEX syndrome

**DOI:** 10.7705/biomedica.7755

**Published:** 2025-09-22

**Authors:** Diego Medina, Camila Ariza-Insignares, Alejandro Restrepo, Ángela Devia, Alexis Franco, Rodrigo Lemus, Manuela Olaya, Rafael Milanés, Harry Pachajoa

**Affiliations:** 1 Unidad Funcional de Trasplantes, Servicio de Hematooncología Pediátrica, Departamento Materno infantil, Fundación Valle del Lili, Cali, Colombia Fundación Valle del Lili Cali Colombia; 2 Facultad de Ciencias de la Salud, Universidad Icesi, Cali, Colombia Universidad Icesi Facultad de Ciencias de la Salud Universidad Icesi Cali Colombia; 3 Centro de Investigaciones Clínicas, Fundación Valle del Lili, Cali, Colombia Centro de Investigaciones Clínicas Fundación Valle del Lili Cali Colombia; 4 Servicio de Endocrinología Pediátrica, Departamento Materno Infantil, Fundación Valle del Lili, Cali, Colombia Departamento Materno Infantil Fundación Valle del Lili Cali Colombia; 5 Unidad de Alergología e Inmunología, Departamento Materno Infantil, Fundación Valle del Lili, Lili, Cali, Colombia Departamento Materno Infantil Fundación Valle del Lili Cali Colombia; 6 Servicio de Gastroenterología Pediátrica, Departamento Materno Infantil, Fundación Valle del Cali, Colombia Departamento Materno Infantil Fundación Valle del Cali Colombia; 7 Servicio de Genética Clínica, Fundación Valle del Lili, Cali, Colombia Fundación Valle del Lili Cali Colombia

**Keywords:** Transplantation, haploidentical, stem cell transplantation, pediatrics, trasplante haploidéntico, trasplante de células madre, pediatría

## Abstract

**Background.:**

IPEX syndrome is a rare hemizygous X-linked disorder with complex autoimmune reactions, characterized by immune dysregulation, polyendocrinopathy, and enteropathy. It has a poor prognosis and a high mortality risk without prompt therapy. Treatment options include pharmacological immunosuppression, nutritional and supportive care, and hematopoietic stem cell transplantation, the latter as the only curative option.

**Case report.:**

We present the case of a male infant, the second child of a non-consanguineous couple, with negative prenatal screening and intrauterine growth restriction detected at 27 weeks' gestation. He was diagnosed with neonatal diabetes mellitus and treated with insulin. He was re-hospitalized for secretory diarrhea and rotavirus infection. At that moment, he was diagnosed with failure to thrive and hypothyroidism. He acquired multiple severe infections, including *Candida parapsilosis* fungemia, an urinary infection caused by extended-spectrum ß-lactamase-producing *Escherichia coli,* and *Klebsiella pneumoniae* bacteremia. Endoscopy biopsy revealed chronic duodenitis with the absence of goblet and Paneth cells, findings suggestive of autoimmune enteropathy. Genetic testing identified a mutation in the *FOXP3* gene, confirming the diagnosis of IPEX syndrome.

We performed a hematopoietic stem cell transplantation from an alternative haploidentical donor and administered a cyclophosphamide post-transplant regime. At 320 days post-transplant, the patient fully recovered his nutritional status and immunity.

**Conclusion.:**

Haploidentical transplantation with a post-transplant cyclophosphamide regime can be a viable therapeutic option for patients with IPEX syndrome, lacking an HLA-identical donor, with promising outcomes based on the follow-up data. Reporting these experiences with haploidentical hematopoietic stem cell transplantation in patients with inborn errors of immunity or other non-neoplastic diseases contribute to the growing body of evidence of this treatment.

## Resumen

**Antecedentes.:**

El síndrome IPEX es un trastorno raro, hemicigótico y ligado al cromosoma X, caracterizado por desregulación inmunitaria, poliendocrinopatía y enteropatía, con pronóstico desfavorable y alto riesgo de mortalidad sin el tratamiento adecuado. Las opciones terapéuticas incluyen inmunosupresión farmacológica, cuidados nutricionales y de soporte, y trasplante de progenitores hematopoyéticos, esta última como única opción curativa.

**Caso clínico.:**

Se presenta el caso de un niño, de sexo masculino, segundo hijo de una pareja no consanguínea, con cribado prenatal negativo y retraso en el crecimiento intrauterino detectado a las 27 semanas de gestación. Se le diagnosticó diabetes mellitus neonatal, que fue tratada con insulina.

El infante fue hospitalizado por diarrea secretora e infección por rotavirus. En ese momento se le diagnosticó retraso del crecimiento e hipotiroidismo. Desarrolló múltiples infecciones graves, como fungemia por *Candida parapsilosis,* infección urinaria por *Escherichia coli* productora de betalactamasas de espectro extendido y bacteriemia por *Klebsiella pneumoniae.* En la biopsia endoscópica se encontró duodenitis crónica con ausencia de células caliciformes y de Paneth, hallazgo sugestivo de enteropatía autoinmune. El análisis genético reveló una mutación del gen *FOXP3,* lo que confirmó el diagnóstico de síndrome IPEX.

Se realizó trasplante de progenitores hematopoyéticos provenientes de un donante haploidéntico alternativo y, luego del trasplante, se administró un esquema de ciclofosfamida. A los 320 días después del trasplante, el paciente recuperó totalmente su estado nutricional y su inmunidad.

**Conclusiones.:**

El trasplante haploidéntico con un esquema posterior al trasplante de ciclofosfamida puede ser una opción terapéutica viable para los pacientes con síndrome IPEX sin donante HLA-idéntico. La experiencia obtenida con este caso mostró resultados prometedores durante el seguimiento del paciente. Estos hallazgos contribuyen a la creciente evidencia sobre el uso del trasplante haploidéntico de progenitores hematopoyéticos en los pacientes con errores innatos de la inmunidad u otras enfermedades no neoplásicas.

The IPEX syndrome (OMIM #304790) is a rare hemizygous X-linked disorder with complex autoimmune reactions, diagnosed in boys typically within the first year of life, characterized by immune dysregulation, polyendocrinopathy, and enteropathy ^1^. Autoimmune reactions resulting in systemic inflammation and metabolic disorders caused by the IPEX syndrome include bone marrow disorders like hemolytic anemia, neutropenia, and thrombocytopenia, associated with splenomegaly. Eczema, refractory diarrhea, and insulin-dependent diabetes mellitus are the three defining clinical features of the syndrome. However, it can also be manifested with other signs, such as autoimmune hepatitis, hypothyroidism secondary to autoimmune thyroiditis, nephropathy, and cardiovascular abnormalities ^2^. These patients are more likely to contract invasive illnesses -such as sepsis, meningitis, pneumonia, and osteomyelitis- and their prognoses are often poor, with a high mortality risk without prompt therapy. Treatment options include immunosuppressive medication, nutritional and supportive care, and hematopoietic stem cell transplantation, the latter as the only curative option ^3^.

IPEX syndrome involves dysfunction of regulatory T cells, decrease CD4+ and CD25+ populations, and uncontrolled proliferation of activated CD4+ effector cells ^3^^,^^4^. This dysfunction results from a mutation of the *FOXP3* gene, located in the short arm of the X chromosome (Xp11.23). The variant alters the structure of the DNA-binding (forkhead, FKH) domain of the scurfin protein -a transcription factor that presumably acts as a pivotal modulator of the regulatory T-cell pathway ^5^^,^^6^.

The diagnosis of the IPEX syndrome might be difficult, but genetic testing can help identify mutations in *FOXP3* with high sensitivity. Thus, the importance of a detailed assessment with high suspicion is key to diagnosis ^7^. Reviewing different case reports, most hematopoietic stem cell transplantation (HSCT) donors were HLA-identical; only one case reported a haploidentical HSCT with T-cell depletion as a rescue therapy after graft failure ^8^. Up to this point, we did not find reports of cyclophosphamide post-HSCT as a curative therapy for IPEX syndrome.

This report presents the case of an infant with IPEX syndrome and mutated *FOXP3,* who underwent a haploidentical HSCT in the absence of an available HLA-identical donor and received cyclophosphamide post-HSCT. This case was reviewed and approved by the institutional review board of the *Fundación Valle del Lili,* Cali, Colombia.

## Case report

We present a male infant, the second child of a non-consanguineous couple, born to a 33-year-old mother, with negative prenatal screening and intrauterine growth restriction detected at 27 weeks' gestation. He had a relevant family history of a brother who died 40 days after birth of an undiagnosed etiology. The patient was born by cesarean section at 37 weeks of gestation, his weight was 1,677 g (SD = -2.84), and his length was 41 cm (SD = -3.23).

He was admitted to the neonatal intensive care unit for nutritional support to promote weight gain. He was diagnosed with neonatal diabetes mellitus and was initially treated with intravenous insulin, later switched to basal-bolus therapy using subcutaneous insulin analogues. After 40 days, he was discharged with adequate metabolic control only supported on bolus insulin.

At the age of three months, he had a second hospitalization due to secretory diarrhea and rotavirus infection. He was diagnosed with failure to thrive and hypothyroidism. He acquired multiple severe infections, including *Candida parapsilosis* fungemia, a urinary infection caused by extended-spectrum ß-lactamase (ESBL)-producing *Escherichia coli,* and *Klebsiella pneumoniae* bacteremia. Subsequently, he was transferred to the *Fundación Valle del Lili* at four months old for multidisciplinary assessment due to chronic secretory diarrhea and suspected diagnosis of metabolic disease, with a Fanconi Bickel syndrome as the first possibility.

Upon admission, the patient's weight was 3,300 g (-6.55 SDS), his length was 57 cm (-3.64 SDS), and weight for height was -5.62 SDS. He looked chronically ill, severely malnourished, and had a giant umbilical hernia, with no other striking findings. Regarding his neurodevelopment, he was at the stage of a two-month-old baby.

In the context of a male infant with a diagnosis of chronic secretory diarrhea, early-onset diabetes mellitus, the previously described family history, and CD4^+^ and CD8^+^ lymphopenia, we suspected an X-linked recessive disorder. Diagnostic studies were ordered to rule out other possible diagnoses. Further investigations included positive thyroid peroxidase (TPO) and thyroid thyroglobulin (TgA) antibodies, suggesting an autoimmune hypothyroidism; antibodies against glutamic acid decarboxylase (GAD) and pancreatic cells (ICA) were not detected.

Endoscopic biopsy reported chronic duodenitis with absence of goblet and Paneth cells suggestive of autoimmune enteropathy. Through analysis of next-generation sequencing of the family DNA, we identified a homozygous variant in the *FOXP3* gene, c.1091A>G (p.Tyr314Cys), classified as likely pathogenic for IPEX syndrome. This finding confirmed the diagnosis.

Regarding treatment, the infant received tacrolimus to control enteropathy, which successfully decreased fecal output. Furthermore, a multidisciplinary assessment concluded that the only curative option was bone marrow transplantation. HLA studies were performed on potential donors. In the absence of an identical donor, the decision was to proceed with a haploidentical HSCT from the father (6/10 HLA match).

Conditioning consisted of fludarabine (1.35 mg/kg) and busulfan (4 mg/kg) on days 5 to 2 before the transplantation, melphalan (2.5 mg/kg on day -1), and thymoglobulin (2.5 mg/kg on days -3 and -2). The infused hematopoietic progenitors came from bone marrow. The patient received 150 *10^7^/kg of nucleated cells, 11*10^6^/kg of CD34+ cells, and 80 *10^6^/kg of CD3+ cells, without complications.

The patient received prophylaxis for hepatic veno-occlusive disease with ursodeoxycholic acid and graft-versus-host disease with cyclophosphamide (50 mg/kg on days 3 and 4 post-HSCT). The patient initiated on day +4 with tacrolimus (adjusted to maintain levels between 8 and 12 ng/ml), combined with methotrexate (7.5 mg/m^2^ on days +5, +7, +10, and +15) and methylprednisolone (0.5 mg/kg from day +5 with tapering on day +30). Additionally, post-transplant antimicrobial prophylaxis included trimethoprim/ sulfamethoxazole, acyclovir, and fluconazole.

Medullary recovery was evidenced on day +14 with neutrophil and platelet engraftment on day +23. Donor chimerism analysis on day +30 showed 99% donor cells in the T-lymphocyte fraction and complete (100%) donor chimerism in whole blood. On day 12 post-HSCT, we identified a catheter-associated infection caused by *Streptococcus mitis* and treated it with ceftriaxone. On day +26, the patient received steroids for suspected cutaneous graft-versus-host disease. His diabetes was closely monitored; despite the need for minidoses of insulin, continuous subcutaneous insulin infusion was deferred due to cutaneous graft-versus-host disease suspicion. However, adequate glycemic control was achieved with basal-bolus insulin analogue therapy.

The patient was discharged 42 days post-HSCT, with tacrolimus and prednisolone as prophylactic management for graft-versus-host disease, and acyclovir, voriconazole, and trimethoprim/sulfamethoxazole as antimicrobial prophylaxis.

During the follow-up, total chimerism showed a progressive decrease with a minimum of 65% on day +180. Thus, immunosuppression was discontinued and, subsequently, the patient improved. On day 320 post-HSCT, total chimerism was 100% (table 1). The patient fully recovered his nutritional status reaching optimal SD for his age, and he also achieved the appropriate level of neurodevelopment.

Regarding comorbidities, the patient remains clinically stable. Secretory diarrhea subsided, and hypothyroidism and diabetes mellitus are under control. He continues on insulin therapy and prophylactic measures. Immunosuppressive medication was administered for mild chronic liver graft-versus-host disease, previously diagnosed due to elevated aminotransferases. This finding was detected during a short hospitalization for an acute respiratory infection, an episode that rapidly responded to a course of calcineurin inhibitor and steroids.

**Table 1:** Patient chimerism following transplantation of hematopoietic progenitors

Type of chimerism	30 days	180 days	320 days
	post-transplant	post-transplant	post-transplant
Donor: recipient complete chimerism in whole blood	100:0	65:35	100:0
Donor: recipient T-lymphocyte chimerism	99:1	98:2	95:5

## Discussion

Immune dysregulation, polyendocrinopathy, and enteropathy X-linked syndrome (IPEX) is an orphan disease with a poor prognosis. During the first two years of life, it has a high mortality risk due to sepsis, metabolic complications, or overall failure to thrive. In the early years, its management can be challenging, often requiring blood transfusions, total parenteral nutrition, dynamic insulin adjustment, immunosuppressive therapy, and prolonged courses of broad-spectrum antibiotics ^8^.

The diagnosis is also challenging due to low suspicion, given its nonspecific symptoms. Genetic assessment is the gold standard to identify *FOXP3* mutations. However, such variants are only identified in 50% of the patients diagnosed with IPEX syndrome. Other genes involved in X-linked dysregulation phenotypes -similar to IPEX - are *STAT5b, STAT1, STAT3, IL2RA, CTLA4, LRBA, TTC7A, TTC37, LRBA, and DOCK8.* Nonetheless, mutations in these genes are not currently recognized as pathogenic ^5^.

In this case report, a diagnosis suspicion of IPEX syndrome arose due to the patient's early-onset polyendocrinopathies with autoimmune characteristics, secretory diarrhea, and failure to thrive; the only typical feature he did not present was eczema. Therefore, this X-linked inborn error of immunity must be suspected in male infants when presenting with the mentioned symptoms, associated with other manifestations such as autoimmune hepatitis, hypothyroidism secondary to autoimmune thyroiditis, nephropathy, and cardiovascular abnormalities. Also, a genetic assessment is required to identify variants associated with the exhibited phenotype ^2^^,^^5^.

In our country, there is only one case reported by Plata-García *et al.,* a toddler with chronic diarrhea, intestinal failure, and recurrent infections. The infant was treated with steroids and calcineurin inhibitors, but died at seven months old with a post-mortem confirmed diagnosis of IPEX syndrome and a genetic analysis confirming a hemizygous mutation in *FOXP3:* c.2T>C (p.Met1Thr) ^9^.

Prompt initiation of immunosuppressive medications can be helpful until HSCT with curative intention. Optimal treatment depends primarily on the availability of a sibling or family donor with identical HLA. In the absence of a compatible donor, the alternative is to opt for an allogeneic HSCT ^10^. A few weeks after the patient arrived at *Fundación Valle del Lili,* he was treated with immunosuppressants, which helped control the fecal output and prevent further weight loss.

Barzaghi *et al.* reported the experience of 96 patients with a genetically confirmed IPEX syndrome from 38 institutions worldwide. Of these, 58 underwent reduced-intensity conditioning HSCT transplantation (33/58). Donor types included HLA-matched donors (52/58), umbilical cord blood (1/58), and haploidentical donors (5/58), most of the latter with α/ß T cell-depleted grafts. The authors did not report the use of cyclophosphamide as a grafft-versus-host disease prophylaxis regimen in this study ^11^. The retrospective analysis showed overall survival of 73.2% post-HSCT (95% CI: 59.4-83.0), and after immunosuppression was 65.1% (95% CI: 62.8-95.8). The study concluded that patients maintained on prolonged immunosuppressive therapy were negatively affected by disease recurrence and complications, which impacted long-term disease-free survival. In contrast, HSCT resulted in disease resolution and a better quality of life ^11^.

For our patient, the only available option was HSCT from a haploidentical donor combined with a cyclophosphamide post-transplant regimen. This graft-versus-host disease prophylaxis strategy has improved recent outcomes in children with other benign diseases undergoing haploidentical HSCT, making it an increasingly most-used therapeutic option worldwide ^12^^,^^13^. To our knowledge, this is the first report on the use of this regimen in IPEX syndrome as a first-line therapy.

Future approaches in gene therapy are promising. Delville *et al.,* performed an *in vivo* assay to assess T_reg_ cell function. The authors, based on adoptive transferthese cells into scurfy mice -an animal model of IPEX- and treated the individuals with a combination of cyclophosphamide and interleukin-2 (IL-2). They demonstrated that the adoptive transfer of FOXP3-transduced scurfy CD4+ T cells enabled the long-term rescue from autoimmune disease ^14^. Until the full development of gene therapies, research on different strategies for HSCT can change the prognosis in these patients.

Based on this case, we consider haploidentical transplantation with a post-transplant cyclophosphamide regime to be a viable therapeutic option for patients with IPEX syndrome, lacking a HLA-identical donor. Our results suggest promising outcomes according to the follow-up data. Reporting these experiences with haploidentical HSCT in patients with inborn errors of immunity or other non-malignant diseases contribute to the growing body of evidence supporting this treatment.
